# Patterns of Ecosystem Metabolism in the Tonle Sap Lake, Cambodia with Links to Capture Fisheries

**DOI:** 10.1371/journal.pone.0071395

**Published:** 2013-08-13

**Authors:** Gordon W. Holtgrieve, Mauricio E. Arias, Kim N. Irvine, Dirk Lamberts, Eric J. Ward, Matti Kummu, Jorma Koponen, Juha Sarkkula, Jeffrey E. Richey

**Affiliations:** 1 School of Aquatic and Fishery Sciences, University of Washington, Seattle, Washington, United States of America; 2 Department of Civil and Natural Resources Engineering, University of Canterbury, Christchurch, New Zealand; 3 Department of Geography/Planning and Center for Southeast Asia Environment and Sustainable Development, Buffalo State, State University of New York, Buffalo, New York, United States of America; 4 Laboratory of Aquatic Ecology, Evolution and Conservation, University of Leuven, Leuven, Belgium; 5 Conservation Biology Division, Northwest Fisheries Science Center, National Marine Fisheries Service, National Oceanic and Atmospheric Administration, Seattle, Washington, United States of America; 6 Water and Development Research Group, Aalto University, Espoo, Finland; 7 Environmental Impact Assessment Centre of Finland Ltd, Espoo, Finland; 8 Finnish Environment Institute, Helsinki, Finland; 9 School of Oceanography, University of Washington, Seattle, Washington, United States of America; Pacific Northwest National Laboratory, United States of America

## Abstract

The Tonle Sap Lake in Cambodia is a dynamic flood-pulsed ecosystem that annually increases its surface area from roughly 2,500 km^2^ to over 12,500 km^2^ driven by seasonal flooding from the Mekong River. This flooding is thought to structure many of the critical ecological processes, including aquatic primary and secondary productivity. The lake also has a large fishery that supports the livelihoods of nearly 2 million people. We used a state-space oxygen mass balance model and continuous dissolved oxygen measurements from four locations to provide the first estimates of gross primary productivity (GPP) and ecosystem respiration (ER) for the Tonle Sap. GPP averaged 4.1±2.3 g O_2_ m^−3^ d^−1^ with minimal differences among sites. There was a negative correlation between monthly GPP and lake level (r = 0.45) and positive correlation with turbidity (r = 0.65). ER averaged 24.9±20.0 g O_2_ m^−3^ d^−1^ but had greater than six-fold variation among sites and minimal seasonal change. Repeated hypoxia was observed at most sampling sites along with persistent net heterotrophy (GPP<ER), indicating significant bacterial metabolism of organic matter that is likely incorporated into the larger food web. Using our measurements of GPP, we calibrated a hydrodynamic-productivity model and predicted aquatic net primary production (aNPP) of 2.0±0.2 g C m^−2^ d^−1^ (2.4±0.2 million tonnes C y^−1^). Considering a range of plausible values for the total fisheries catch, we estimate that fisheries harvest is an equivalent of 7–69% of total aNPP, which is substantially larger than global average for marine and freshwater systems. This is likely due to relatively efficient carbon transfer through the food web and support of fish production from terrestrial NPP. These analyses are an important first-step in quantifying the resource pathways that support this important ecosystem.

## Introduction

The accumulation and processing of carbon (C) and energy within ecosystems has been a dominant theme in ecology for much of its history and is an important determinant of ecosystem functioning (sensu [1]). Traditionally in aquatic ecosystems, the focus has been on *in situ* primary production as the primary energy source for ecosystems and food webs. However, beginning in small streams, then large rivers and eventually lakes, it is now recognized that energy and organic matter are often transferred across ecosystem boundaries – between the aquatic and terrestrial landscape – through multiple pathways, and that heterotrophic processing of organic matter is a potential source of carbon and energy to upper trophic levels [2], [3], [4]. Despite the general awareness of autotrophic and heterotrophic pathways of energy flow within food webs, knowledge of their importance for overall secondary productivity among ecosystems remains relatively rudimentary. Studies of aquatic ecosystem metabolism – rates of gross primary productivity and ecosystem respiration – describe the overall magnitude and relative balance of heterotrophic and autotrophic processes within an ecosystem [5], and are therefore extremely useful in understanding critical ecosystem processes such as energy flows, nutrient cycling, carbon balance, trophic state, and food web dynamics [6].

Compared to temperate and high-latitude lake and stream ecosystems, far less is known about ecosystem functioning in tropical freshwater ecosystems. Many tropical lakes and rivers have broad floodplains which are connected to permanent water through seasonal flooding. Floodplains are inherently dynamic and cannot be easily categorized as aquatic or terrestrial, but instead are defined by seasonal changes in the hydrologic environment. These “flood-pulse” ecosystems [7] alternate in their functioning between a primarily aquatic-phase and primarily terrestrial-phase, with each influencing the other (e.g., through sediment deposition or organic matter production).

**Figure 1:**
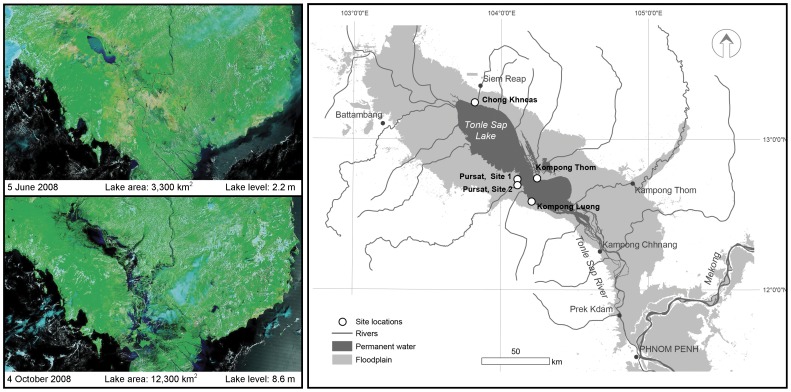
MODIS satellite imagery of the Tonle Sap ecosystem during the dry (top panel) and wet (bottom panel) seasons in 2008. Lake area estimates corresponding to the satellite image date were derived from the hydrology model presented in Kummu et al. [49]. Lake level data are in m a.s.l. relative to the Ha Tien datum. The lake bottom is approximately 0.5–0.7 m a.s.l. (right) Map of sampling sites in the Tonle Sap lake and floodplain.

The Tonle Sap Lake in Cambodia is a flood-pulse ecosystem within the Lower Mekong River basin of Southeast Asia (Figure 1). The river, lake, and floodplain are hydro-dynamically connected via the Tonle Sap River where, during the dry season (November-July), the lake drains via the Tonle Sap River into the Mekong near the Cambodian capital of Phnom Penh. For much of its length, the lower Mekong remains one of the last unregulated great tropical rivers, and with the annual rise of the river during the wet season, the Tonle Sap River reverses its flow direction to fill the lake with water and sediments. Lake levels rise from an average of 0.8 to over 9 meters and the areal extent of the lake increases by more than five-fold (roughly 2,500 km^2^ to >12,500 km^2^), extending surface water into the broad surrounding floodplain vegetation (Figure 1). The Mekong is currently facing rapid hydroelectric dam development, with plans for twelve mainstem and over 70 tributary dams by five of the six countries that border the river [8], [9], [10]. These projects would modulate the flood-pulse of the river [11], [12] and downstream sediment delivery [13] with potentially large ecological effects.

The importance of determining sources and fates of carbon to ecosystems is elevated when livelihoods depend on these resources (e.g., fisheries), and when future environmental change – anthropogenic or otherwise – threatens to alter ecosystem dynamics related to ecosystem function. The Lower Mekong Basin supports one of the largest freshwater capture fisheries in the world [14]. This fishery is critical to the people of mainland Southeast Asia, supplying much of their animal protein, calcium, and vitamin A, and is an important driver of local economies and culture of area residents [15]. Despite its importance, there is relatively little primary ecological information for the Mekong and Tonle Sap, particularly with respect to ecosystem-scale dynamics that contribute to fish production and local livelihoods. In particular, there is currently no direct information on aquatic primary productivity in the lake and floodplain and how primary production relates to overall fisheries production. Similarly, there is no direct knowledge about heterotrophic processing of organic matter in the lake and the potential for fish production through bacterial pathways (i.e., the microbial loop). While there are many reasons to expect strong links between the flood-pulse hydrology of the system and important ecosystem functions, these links have yet to be established directly either through experimentation or observation.

Here we present the first measurements of aquatic ecosystem gross primary productivity (GPP), respiration (ER), and metabolic balance (ER/GPP) from the Tonle Sap lake and floodplain. We used an inverse modeling approach to calculate ecosystem metabolic rates from continuous records of dissolved oxygen (DO) at four sites. The model was developed in a Bayesian state-space framework that considered prior information on air-water gas exchange and errors in both observation and process when fitting the data. State-space models have become highly relevant for modeling environmental data, because they link observed data to the underlying natural processes that are never seen directly due to observation and process uncertainty. We used the resulting metabolism estimates to calibrate a three dimensional hydrodynamic-productivity model to calculate aquatic net primary production (aNPP) and compare annual carbon fixation with carbon removal through the fishery.

## Methods

### Continuous DO Data Collection

We used data sondes to collect continuous records of turbidity, dissolved oxygen, conductivity, and temperature at four locations in the Tonle Sap (Table 1, Figure 1). The longest record was from a site at the northern end of the lake near the floating village of Chong Khneas. This site was in close proximity to emergent vegetation (∼1 km) but had open water throughout the sampling. The second location was within the seasonally flooded gallery forest in Pursat province on the southwestern shore of the lake. Midway through the sampling at Pursat the data sonde was moved 4.5 km southwest to track the expansion of the lake during the flood-pulse. The third sampling site was in the open water near the village of Kompong Luong. The fourth site was in the flooded forest at the opposite shore of the lake from Kompong Luong in Kampong Thom province. All necessary permissions for the described field studies were obtained from the Royal Cambodian Government and the Mekong River Commission. More specific details about data collection and site details can be found in Irvine et al. [16] and Sarkkula et al. [17].

**Table 1:** Location and sample collection information.

Locations	Latitude (DD)	Longitude (DD)	Dates	Water depth* (m)	Sample depth (m)
Kompong Luong (open water)	12.5849	104.2104	21 October 2001–12 November 2001	7.1–8.1	1.5
Kompong Thom (flooded forest)	12.7393	104.2470	21 October 2001–12 November 2001	5.8–6.8	3.5
Pursat, Site 1	12.7333	104.1138	1 August 2005–26 August 2005	2.0–4.9	1.4
Pursat, Site 2	12.6937	104.1137	10 September 2005–3 November 2005	5.7–6.8	1.4
Chong Khneas	13.2429	103.8229	1 September 2007–31 March 2009	1.1–7.7	0.5

* given as the range over the sampling interval.

Sondes were placed within what was believed to be the surface mixed layer. Temperature profile data were not available during the time of sampling, but previous and subsequent sampling shows vertical temperature change of 1°C or less at most locations [18], [19]. Generally conditions during the wet season were isothermal at dawn with warmer surface waters by late afternoon and thermoclines, when developed, at approximately 2 m. During the dry season conditions remained isothermal. Sondes were set to record data at 30 min intervals. Routine servicing of the rapid-pulse dissolved oxygen probes to replace the membrane and electrolyte solution occurred approximately every two weeks creating small breaks in the data. On three occasions the equipment at the Chong Khneas site either failed or was improperly calibrated. In total there were 475 days of usable data from the Chong Khneas site, 82 days from the Pursat site, and 23 days from the Kampong Luong and Kampong Thom sites.

### Bayesian Estimation of Ecosystem Metabolism from Continuous DO Data

High resolution, continuous DO and water temperature data were analyzed in a dynamic aquatic ecosystem metabolism model similar to the Bayesian Metabolic Model presented in Holtgrieve et al. [20]. This model simulates diel patterns in DO for each individual day in the data set as a function of gross primary production (*GPP*), ecosystem respiration (*ER*), and O_2_ exchange with the atmosphere (*G*). The time rate of change in DO concentration ([O_2_], in mg L^−1^) is a first-order differential equation of the form:
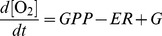
(1)


*GPP* (mg O_2_ L^−1^ t^−1^) was modeled as a linear function of light.
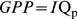
(2)where, *t* is the time step between data points (30 mins), *I* is the estimated photosynthesis-irradiance coefficient (mg O_2_ L^−1^ t^−1^)/(mmol photons m^−2^ s^−1^) and Q_p_ is the photosynthetically active photon flux density (mmol photons m^−2^ s^−1^). Q_p_ was modeled using a geographically-based light model [20] that assumed uniform atmospheric transparency. Ecosystem respiration at a standard 20°C (*ρ_20_*, in mg O_2_ L^−1^ t^−1^) was estimated by the model and adjusted to *in situ* water temperature (T) using the following Van’t Hoff- Arrhenius relationship.



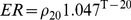
(3)Finally, atmospheric exchange of O_2_ was modeled as the gradient between the *in situ* dissolved oxygen concentration and the concentration at atmospheric equilibrium ([O_2,sat_]), multiplied by an estimated reaeration coefficient for O_2_ (*K*, in units of t^−1^).
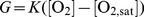
(4)


*K* was converted to a standardized 20°C (*K_20_*) using *in situ* water temperature and Schmidt numbers [21].

Errors in model fits to data can arise from multiple sources including errors in measurement (observation error), inherent stochasticity (process error), and the inability of simplified models to describe complex ecological processes (model errors), among others [22], [23]. We used a Bayesian linear state-space model, similar to a Kalman filter, to assess both observation and process uncertainty in model fits to the data [24]. The state (*X*) is estimated as a separate autoregressive process for each day, with initial conditions estimated as unknown parameters (the first states, *X_day,0_*). Given a set of oxygen model parameters and equations – represented in Eq. 5 by a linear model and the parameters *m* and *b* – and an estimated process deviation (*p_day_*), the unseen ecosystem condition is *X_day,i_*, in this case the true [O_2_] for each successive time step (*i*) on a given day.
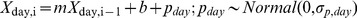
(5)


These unseen states are then linked to the observed data (*Y_day,i_*, the measured [O_2_] at each time step) through the observation equation with an associated observation uncertainty (*o_day,i_*). Each day is allowed to have a unique process variance allowing some days to be more stochastic than others.
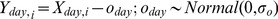
(6)


Under a Bayesian context, prior information on model parameters was supplied to the model as probability density functions and evaluated together with observed data. Parameters *I*, *ρ_20_,* and starting condition ([O_2_] at sunrise) were given uniform priors across a wide range of possible values with equal probability. At the Chong Khneas site, *K_20_* was given normally distributed priors for each day with mean and standard deviation derived from daily average wind speed data and the empirical relationship predicting *k_600_* (the gas transfer velocity in cm h^−1^ at a Schmidt number of 600) as a function of wind speed at 10 m in Ho et al. [25]. Daily average wind speed data were from the Global Surface Summary of Day (GSOD) for the Siem Reap NOAA National Climatic Data Center (NCDC) meteorological station (station 48966) located approximately 15 km from the Chong Khneas site (Figure 1). Wind speed based estimates of *k_600_* were converted to *K_20_* using Schmidt numbers and an assumed vertical mixing depth of 0.5 meters. Wind speed data were not available for the time period of the Pursat or Kampong Luong sampling. We therefore assigned *K_20_* a uniform prior from 0 to 4 h^−1^. Because of the autoregressive nature of our model (eqn [5]), we were able to estimate the mean and variance of the underlying state for each day in the time series; stochasticity was treated as temporally independent, and with a constant variance (*σ_p_*). Similarly, observation error variance (*σ_o_*) was estimated as a single parameter shared among all days for a given site.

Posterior probability distributions for model parameters (*I*, *ρ_20_*, *K_20_*) where estimated for each day in the data set using Markov Chain Monte Carlo (MCMC). This method creates a linked series of model estimated parameter values which have high likelihood given the observed data and the prior information provided to the model. Parameter values for each saved draw in the MCMC chain were used to calculate ecosystem metabolism (GPP and ER) at each time step and summed into daily rates using equations 2 and 3. We implemented our model using the JAGS (Just Another Gibbs Sampler) software package interfaced to R via the runjags() and R2jags() packages [26], [27], [28]. Posterior predictive checks for observed versus predicted values were used to diagnose and assess the quality of model fits to the data. Three replicate MCMC chains were run with a minimum of 35,000 iterations, a 20,000 iteration burn-in, and thinned by half for a total of 7,500 saved draws. Posterior distributions were tested for autocorrelation among saved draws using the ‘acf’ function in R, the lack of which indicates convergence of model estimates on the underlying true parameter distribution. The posterior distributions are presented as the median, 2.5% and 97.5% credible limits of the saved MCMC chains.

### Proportion of Whole-lake aNPP Removed by the Fishery

To compare the amount of carbon moving through the trophic web to ultimately be extracted by the fishery with total aNPP, we used a previously developed spatially explicit EIA 3-dimensional hydrodynamic-productivity model [29], [30]. The model predicts planktonic and attached (periphyton) aNPP spatially as a function of euphotic volume, euphotic duration, and primary productivity estimates from the literature (see [30] for specific values). We used our estimates of GPP from the Bayesian model to further calibrate the EIA model for the sites and dates where we had data. To convert from gross to net primary productivity, we applied a photosynthetic coefficient of 1.2 and assumed net was 60% of gross before converting from oxygen to carbon units using their molecular mass [31], [32]. We then randomly divided our estimates of net primary productivity (at the 50% credible limit) into individual calibration and validation data sets. Using the calibration data set, we adjusted model primary productivity coefficients by minimizing the sum of squares between the observed and model predicted values. Once the model was calibrated, its ability to predict aquatic NPP was assessed using the validation dataset. For the calibration dataset, the mean squared error (MSE) was 0.41 and the average match was 81±48%. For the validation dataset, the MSE was 0.38 and the average match was 76±46%. Finally, the calibrated model was used to estimate total annual aNPP (tonnes C y^−1^) for the entire lake and floodplain for the years 1997–2008, the date range when the necessary hydrologic data was available to parameterize the model.

Fish biomass removed by the fishery was converted to equivalent units of NPP and compared with total annual aNPP (*sensu* [33]). Total biomass removed by the fishery was converted to NPP by the following equation:
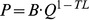
(7)where, *P* is the primary production that has been converted to fish biomass (in tonnes C y^−1^), *B* is the total annual catch (tonnes C y^−1^), *Q* is the efficiency with which carbon is transferred between trophic levels, and *TL* is the average trophic level of the catch. Total biomass was converted from fresh weight to carbon units assuming a wet to dry mass ratio of 4 and 44.6% carbon content. The specific values of *Q* and *TL* for the Tonle Sap fishery are not well known. *Q* is commonly estimated at 0.1 (10% transfer of carbon between trophic levels) based on the data compilation in Pauly & Christensen [33]. To estimate *TL*, the believed top ten fishery species by total catch [34] were assigned minimum and maximum trophic levels from FishBase [34], [35] which allowed us to calculate an average trophic level of the Tonle Sap catch to be between 2.3 and 2.7. We produced a set of four scenarios with various combinations of 0.1 and 0.15 for *Q* and 2.3 and 2.7 for *TL* over a range of potential fisheries yields (150,000 to 300,000 tonnes y^−1^).

## Results

### Seasonal Dissolved Oxygen Dynamics

**Figure 2:**
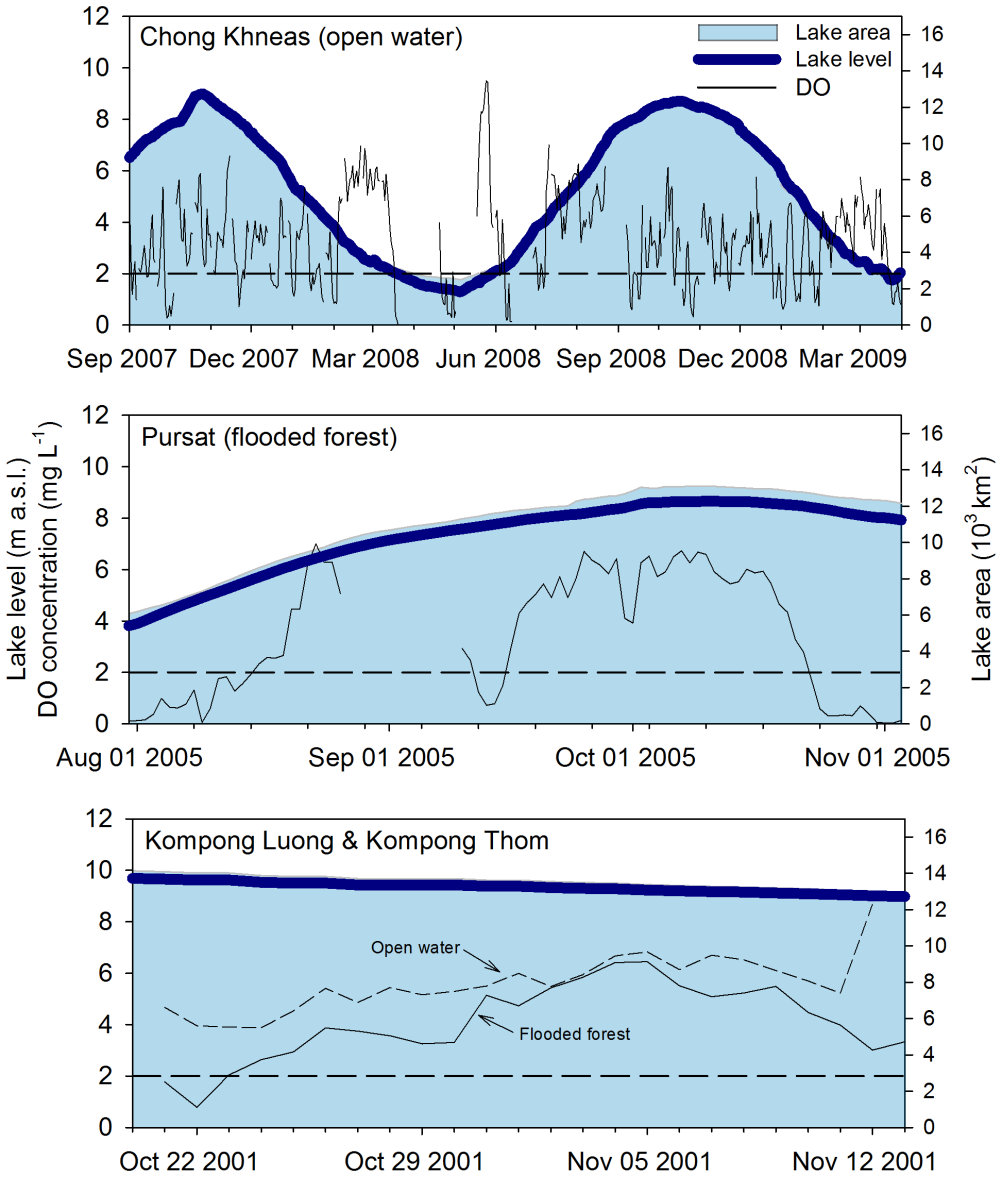
Continuous records of daily average dissolved oxygen concentration in the Tonle Sap lake and floodplain. The horizontal dashed line at 2 mg L^−1^ indicates hypoxic conditions at the sampling depth. Also shown are lake level and modeled lake area as described in Figure 1.

Average daily DO concentrations at all the sites in the Tonle Sap were highly variable, with values from 9.5 mg L^−1^ (127% of saturation) to complete anoxia, and showed little pattern with season and water level (Figure 2). The vast majority of days (86%) had DO concentrations consistently below 100% of saturation, indicating generally net heterotrophic conditions where ER exceeds GPP. Hypoxic conditions (DO <2 mg L^−1^) were also frequently observed irrespective of location, season, or flood extent. At the Chong Khneas site with the longest continuous record, daily average DO was <2 mg L^−1^ on 121 of 475 days (25%). There were more than 300 days (63%) with hypoxic conditions for at least part of the day, usually at night when there is no photosynthetic production of oxygen. With the exception of the open water site at Kompong Luong, hypoxic conditions were observed at all sites where we had data.

### Estimates of Ecosystem Metabolic Conditions

Of the 727 total days for which we had DO data, 269 had sufficient diel changes in DO to robustly estimate GPP and ER. For each day we estimated a probability distribution for the most likely values of ER and GPP given the data, the assumptions of our model, and any prior information (e.g., gas transfer). Here we present the median of the posterior distribution as the best point estimate of ecosystem metabolism for a given day.

The average values of GPP and ER across all sites and all days were 4.1±2.4 and 24.4±19.9 g O_2_ m^−3^ d^−1^ respectively. There were substantial differences in average ER among sites, but relatively little difference in GPP (Table 2). The Chong Khneas open water and Kompong Thom flooded forest sites had estimates of ER that were six to nine times greater than the other two sites; there was no pattern in ER between the flooded forest and open water sites. In contrast, average GPP for the Chong Khneas, Pursat, and forest site at Kompong Thom were very similar, with the open water site at Kompong Luong about 40% lower.

**Table 2:** Average GPP and ER at four locations within the Tonle Sap ecosystem.

		ER	GPP	aNPP*	n days
		(g O_2_ m^−3^ d^−1^)	(g O_2_ m^−3^ d^−1^)	(g C m^−3^ d^−1^)	
Chong Khneas					
(open water)	Jan	34.9±1 5.2	4.5±2.7	0.8±0.5	32
	Feb	22.1±11.6	4.7±1.8	0.9±0.3	31
	Mar	32.5±21.1	5.7±3.0	1.1±0.6	36
	Apr	NA	NA	NA	NA
	May	NA	NA	NA	NA
	Jun	14.4±11.2	3.3±2.3	0.6±0.4	3
	Jul	13.9±6.1	4.3±2.5	0.8±0.5	15
	Aug	14.4±8.3	2.5±1.2	0.5±0.2	9
	Sep	34.4±13.2	4.9±2.7	0.8±0.2	23
	Oct	34.0±31.9	3.6±2.1	0.9±0.5	24
	Nov	35.5±18.9	2.9±1.7	0.7±0.4	28
	Dec	22.1±18.3	2.6±1.7	0.5±0.3	22
	Mean	28.9±19.5	4.2±2.5	0.5±0.3	223
Pursat					
(flooded forest)	Aug	3.4±1.0	3.1±0.7	0.6±0.1	4
	Sept	4.2±1.8	4.1±0.9	0.8±0.2	12
	Oct	4.9±2.2	4.0±0.9	0.8±0.2	17
	Mean	4.0±0.9	4.4±1.10	0.7±0.2	33
Kompong Luong					
(open water)	Oct & Nov	2.9±1.4	2.6±0.8	0.5±0.1	10
Kompong Thom					
(flooded forest)	Oct & Nov	26.7±13.7	4.3±0.9	0.8±0.2	3

* aNPP calculated from GPP assuming that autotrophic R is 40% of GPP and a photosynthetic quotient of 1.2.

**Figure 3:**
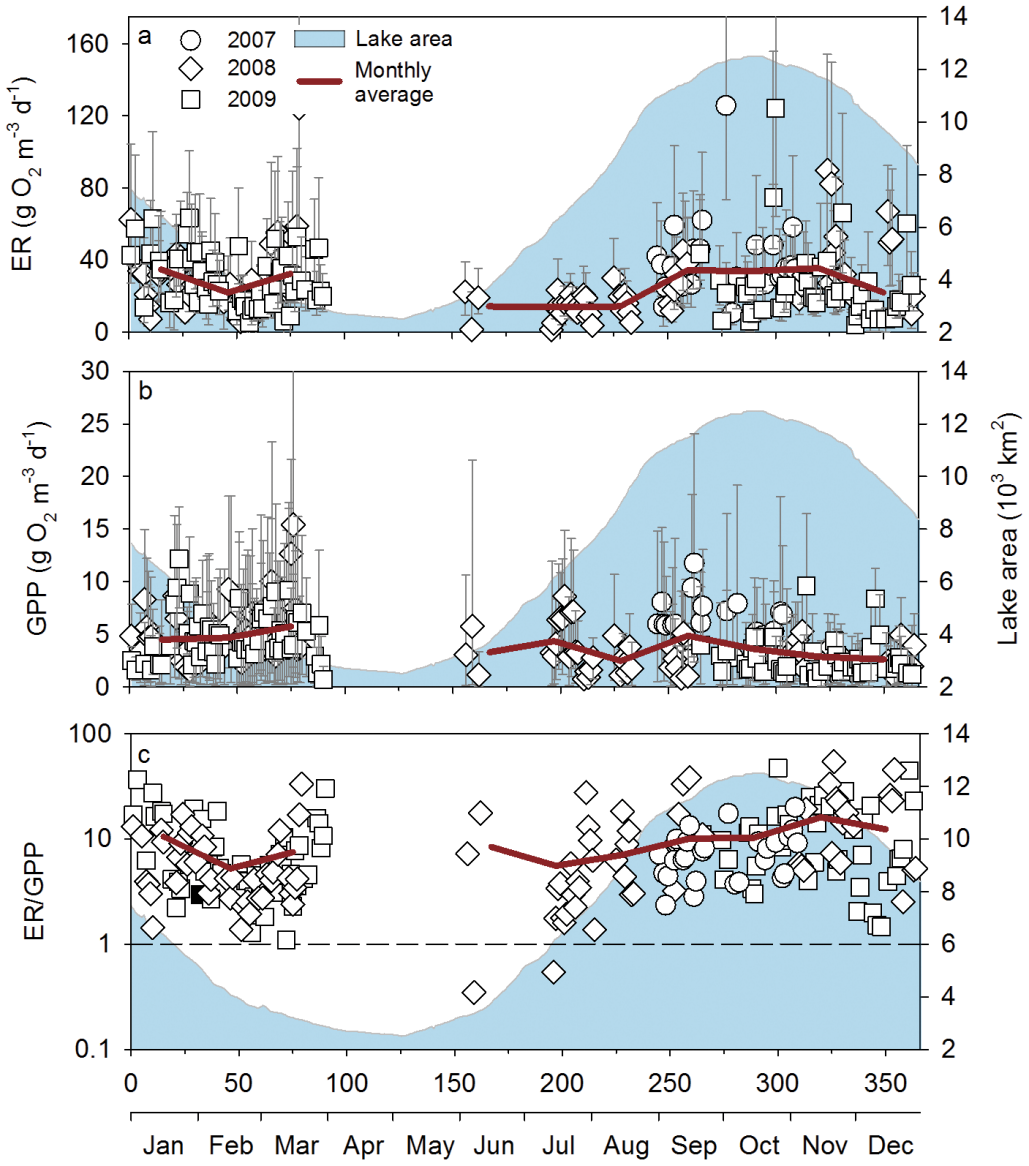
Ecosystem metabolism estimates for the Chong Khneas site (9/2007 to 4/2009). Data points are daily estimates from the Bayesian state-space model thinned to only include days where diel changes in oxygen were sufficient to produce reliable values. Each point is the median of the posterior distribution for a given day with error bars indicating the 95% credible interval. The red lines are the monthly mean. Also shown is modeled lake area as described in Figure 1.

The nearly year and a half record of DO at the Chong Khneas site allowed for inferences on seasonal changes in ecosystem metabolism (Figure 3). At this location there was a general trend for increased GPP as the water level declined in the lake with the highest daily and monthly average values in March and the lowest in August, November, and December. Seasonal patterns in ER also varied over the hydrologic cycle. The highest daily and monthly averages were in September to January corresponding to high and falling water; however, March also showed high ER when the water level was approaching the annual minimum. The overall seasonal range in ER at this one site eclipsed the spatial differences among our four sites. Unfortunately we were unable to fully characterize the complete seasonal change in GPP and ER from high to low water due to equipment failure at the Chong Khneas site and lack of low water sampling at the remaining sites.

### Relationships between Metabolism and Physical Conditions

**Figure 4:**
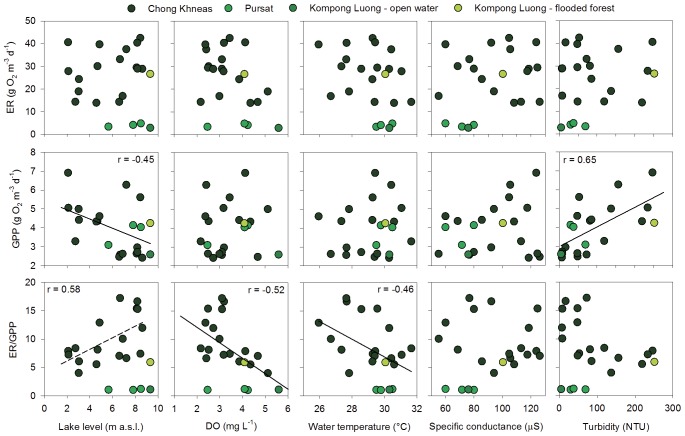
Correlations between Tonle Sap ecosystem metabolic properties and physical drivers. Data points are monthly averages for each site and year. Dissolved oxygen, water temperature, specific conductance, and turbidity are from concurrent measurement at each site using data sondes. Lines indicate significant Pearson’s correlations following a Student's t-distribution (α = 0.05). The dashed line indicates that only data from the Chong Khneas site were included in the correlation analysis.

We compared rates of ER, GPP and the GPP:ER to five parameters describing the physical condition of the lake: water level, average DO concentration, water temperature, specific conductance, and turbidity. Both ecosystem metabolic rates and physical parameters were synthesized into monthly averages. Average ER was not correlated with any of the physical parameters (Figure 4). Average GPP, on the other hand, was negatively related to lake level and positively related to turbidity. The ratio of ER to GPP was negatively related to average DO and water temperature. In general, these patterns were consistent whether the longer record at the Chong Khneas site was examined alone or including all the sites together. The two exceptions are that ER/GPP was positively related to lake level and negatively related to turbidity when looking at the Chong Khneas data alone. ER and GPP were not correlated as has been previously found in a number of other ecosystems [36]. We also considered the same set of relationships using daily metabolism estimates (data not shown) and found weak but significant relationships between GPP and turbidity (Kendall τ  = 0.27), ER and water temperature (τ = -0.20), ER and average DO (τ = -0.45). ER/GPP followed the same relationships as ER and was correlated with water temperature (τ = -0.24) and average DO (τ = -0.50).

### Calibration of Aquatic Net Primary Production Model

Using our site-based measurements of aquatic primary productivity from 269 sampling days to calibrate the hydrodynamic-productivity model, we estimated an annual lake and floodplain combined aNPP for the years 1997–2008 ranging between 2.1 to 2.7 million tonnes C y^−1^ with an average of 2.4±0.2 million tonnes C y^−1^. Average areal rates of aquatic net primary productivity for both the permanent lake and active floodplain ranged from 1.7 to 2.4 g C m^−2^ d^−1^ among years with an overall average of 2.0±0.2 g C m^−2^ d^−1^.

### Extraction of NPP by Fisheries

Unfortunately data on total fisheries catches from the Tonle Sap are limited. Official estimates of fisheries range from 230,000 to 250,000 tonnes fresh weight annually [37], however Lamberts [38] questions the validity of these estimates given the lack of comprehensive monitoring data. In contrast, household surveys of fish consumption suggest that commercial and subsistence harvest from the lake could be as high as 290,000 tonnes [15]. We therefore conducted our analyses over a range of hypothetical fisheries yields from 150,000 to 300,000 tonnes.

**Figure 5:**
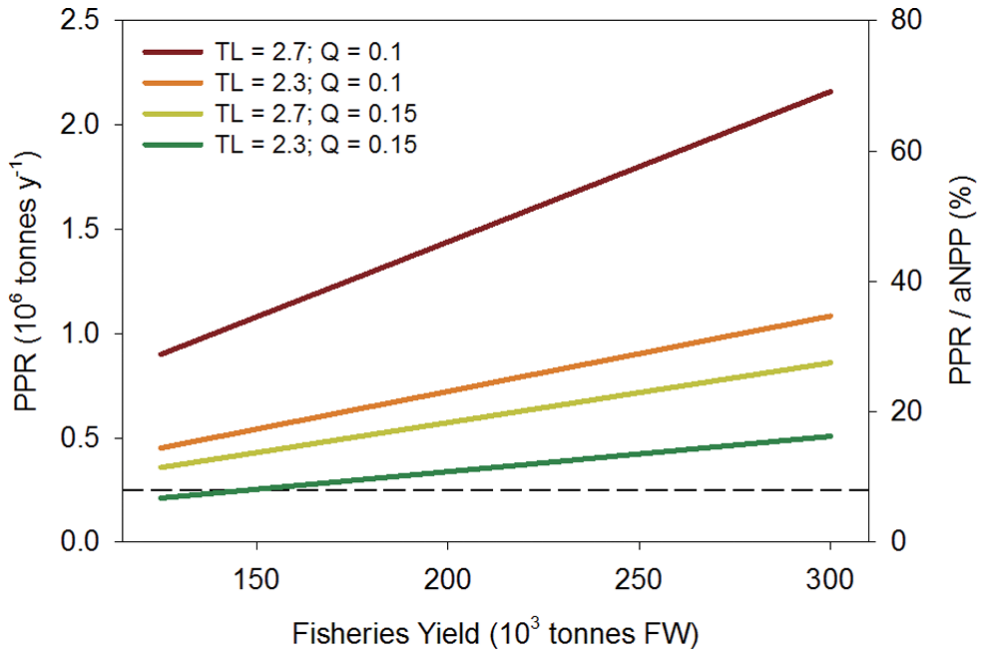
The amount of primary production required to support the Tonle Sap fishery (PPR) over a range of potential fisheries yields (see text for explanation). Each line is a different scenario of carbon transfer through the food web and is a function of the average trophic level of the catch (*TL*) and trophic efficiency (*Q*). The right axis shows PPR as a percent of total aquatic net primary production (aNPP). The dashed line indicates the global weighted average for freshwater and marine fisheries (∼8%) [33].

Applying equation 7 and assuming a system highly efficient at transferring carbon through the food web – average *TL* of 2.3 and trophic efficiency (*Q*) of 0.15 – the amount of primary production captured in fisheries (PPR) was approximately 8 to 16% of total aNPP (Figure 5). In contrast, with a relatively inefficient system with respect to carbon transfer (*TL* = 2.7 & *Q* = 0.1) fisheries extraction would amount to 35% to 69% of aNPP. For the two middle scenarios and Tonle Sap fisheries production of 250,000 tonnes and above, fisheries production as a percent of aNPP exceeds the global average of 24% for freshwaters and all but the most productive marine ecosystem [33]. These values do not include the spawning stock of fish that remain in the lake or fish lost from the system through emigration, non-fish predation (i.e., birds), or natural mortality. Combined these could easily increase the required amount of primary production for fish by 50% or more.

## Discussion

### Oxygen Dynamics and Ecosystem Metabolism

Continuous monitoring of dissolved oxygen and water quality parameters provides valuable data for interpreting ecological conditions and evaluating ecosystem functions not possible with spot measurements alone [16], [20]. The widespread and repeated hypoxia observed in the Tonle Sap lake and floodplain (Figure 2) is most likely a natural phenomenon due to a combination of high water temperatures leading to low oxygen solubility and organic matter inputs to the aquatic environment from both the Mekong River and the surrounding floodplain. Organic matter inputs from outside of the aquatic ecosystem fuels *in situ* consumption of oxygen via heterotrophic respiration in excess of oxygen production. Bacterial metabolism of detritus likely brings some of this material into the aquatic food web and is most efficient under oxic conditions. When oxygen is depleted, anaerobic metabolism of carbon is another mechanism to move carbon from the detrital pool into living biomass. This pathway is far less efficient than aerobic pathways since energy in the form of reducing power is transferred to non-carbon elements (i.e., H_2_S, NH_4_^+^, Fe^2+^) or to methane that can escape the ecosystem [39]; although, if methane is subsequently oxidized by methanotrophs, that energy remains within the ecosystem. Significant losses of carbon via non-oxidative pathways also results in an underestimate of total heterotrophic respiration (aerobic+anaerobic) using the oxygen method.

Our estimates of ER by oxygen mass balance were large compared with other studies and variable in both space and time (Table 2). For example, Solomon et al. [36] calculated pelagic ER from continuous oxygen records for 25 globally distributed lakes and found that peak rates ranged from 0.4 to 20 g O_2_ m^−3^ d^−1^. Peak rates for the Chong Khneas site were as high as 150 g O_2_ m^−3^ d^−1^ and the mean for all days at this site was 29±20 g O_2_ m^−3^ d^−1^ (Table 2). High rates of ER are not surprising for a large, warm and shallow lake with strong flood-pulse interactions and highly productive floodplain vegetation [38]. However, the sensor for this site was placed off a floating building near a seasonally migratory village, and therefore may be more reflective of local-scale conditions directly influenced by humans than average conditions across the lake. Similarly, the sensor at the Kampong Thom site (ER of 27±14 g O_2_ m^−3^ d^−1^) was placed relatively deep in the water column and likely captured benthic respiration to a greater degree than typical pelagic sampling near the water surface [40].

At all sites the aquatic component of the Tonle Sap ecosystem was dominantly net heterotrophic with GPP exceeding ER on only two of our sampling days (Figure 3). There was limited coupling of ER and GPP as is commonly found across all lake types, but this is generally strongest in oligotrophic or mesotrophic ecosystems [36]. ER was also poorly correlated with any of the physical drivers we tested (Figure 4). Frequent hypoxia, strong heterotrophy, and lack of correlation between ER and GPP suggests that a large amount of organic carbon is being processed through microbial pathways in the lake, and the low rates of GPP relative to ER and secondary production suggest much of this organic carbon is allochthonous (i.e., terrestrial NPP).

Site-based differences in ER appeared to be related to position within the basin. Higher rates of ER were at the sites on the northern shore (Chong Khneas & Kompong Thom), while lower ER occurred at sites on the southern shore (Pursat & Kompong Luong; Table 2). The northern floodplain near Kompong Thom extends approximately 30 km from the permanent lake and the Chong Khneas site sat at the base of a large floodplain extending to the northwest (Figure 1). In contrast, the floodplain near the Pursat and Kompong Luong sites extends roughly 3 km from the lake edge. Therefore the site differences in ER may be related to the effective “recruitment area” for terrestrial organic matter for any given location within the lake. A second factor influencing ER may be the timing within the flood cycle. The Pursat sites were sampled during rising water near the edge of the flooded area, and the low ER values relative to the other sites may be due to the terrestrial organic matter needing time to condition before microbial decomposition. Unfortunately, however, we cannot distinguish between the potential role of organic matter and flood timing in controlling ER and possible confounding effects of humans and vertical placement of the sensors discussed above.

Lamberts and Koponen compiled data from 51 tropical flood-pulsed lakes with phytoplankton productivities ranging from <0.001 to 17.9 g C m^−3^ d^−1^ [30]. A similar survey of specifically large lakes (>500 km^2^) worldwide provided a range of primary productivity from 0.01 to 7.2 g C m^−2^ d^−1^ with a mean among tropical lakes of 2.6±2.1 g C m^−2^ d^−1^ [41]. Our average net primary productivity estimate for the Tonle Sap of 0.8±0.4 g C m^−3^ d^−1^ (2.0±0.2 g C m^−2^ d^−1^) falls in the middle of these ranges, which is not surprising since the lake is generally productive but does not suffer from significant cultural eutrophication as do many high-productivity lakes. The inter-annual variation and variation among sites in GPP was also small relative to the range of values from the literature both within and among lakes. However, as noted above, our sampling misses what is likely the time of maximum primary productivity during most of the low-water period. Also, surface blooms of blue-green algae are common during low-water [42] and their contribution to GPP would likely not be fully captured by oxygen mass balance due to direct exchange with the atmosphere. Finally, two of our four sites were near sizable local villages and it is possible that human wastes could influence local dissolved oxygen and ecosystem metabolism, however no clear pattern was apparent.

Despite missing two of the low water months, we observed a significant negative correlation between GPP and lake level and a positive relationship with turbidity (Figure 4). This could be due in part to the concentration of phytoplankton and nutrients as water volume declines [42]. The positive relationship with turbidity could reflect higher phytoplankton biomass or the sensor could be influenced by resuspended sediments from the bottom of the lake as water depth declines. Future work coupling GPP measurements, phytoplankton biomass (chlorophyll-a), and suspended sediments would better constrain factors influencing GPP.

The factors limiting primary production in the Tonle Sap are currently unknown but of critical importance to understanding the future of the lake. At short time scales (i.e., daily), the amount of light penetrating into the water column generally limits photosynthetic production [43]. It is currently unknown however, the extent to which light, nutrients, or grazing limits primary production and algal biomass in the Tonle Sap over longer time scales. The flood-pulse nature of the ecosystem provides a mechanism to transfer nutrients between the river and the lake or the lake and the floodplain. It has generally been assumed that reduced sediment inputs to the lake due to trapping by proposed upstream hydroelectric dams will decrease primary productivity in the Tonle Sap through decreased inputs of limiting nutrients, although this has not been tested either directly or indirectly. It is also unknown if the limiting nutrient is nitrogen, phosphorus, or micronutrients, and it is also conceivable that light is the primary limiting factor for algae in this system. Understanding which specific physical, chemical, or biological factors control aquatic photosynthesis is a critical next step in determining the likely impacts of dam construction on primary production.

It should be noted that the continuous records of DO we used to estimate ecosystem metabolism were not necessarily initiated with this goal in mind, and therefore limits the inferences that can be made. Although GPP appears not to vary substantially among these sites, the temporal sampling is limited and there is reason to expect higher spatial variation at other times of year. Similarly, ER is also likely to show habitat-specific variation when considering different periods in the flood-pulse. In general, the hydrodynamic-productivity model we used to scale our NPP estimates under-estimated the measured values based on DO mass balance: measured NPP was 0.89±0.53 g C m^−2^ d^−1^ while the model predicted 0.52±0.13 g C m^−2^ d^−1^ for the same locations and dates of the measurements. To fully constrain ecosystem productivity – the ultimate basis for fish production – will require broader spatial and temporal sampling for metabolism and adapting the existing models to capture this variation in both space and time. It will also require direct information of periphyton and macrophyte productivity, and transfer of terrestrial resources to the aquatic food web.

### Connections to Fisheries

Fishing activities in the Tonle Sap are ubiquitous and distributed among thousands of commercial and subsistence fishers. The diffuse nature of the fishery makes monitoring of catches over the long-term exceedingly difficult, if not impossible. However, considering a wide range of possible fisheries harvests, the amount of carbon leaving the system is between 7% and 69% of aNPP (Figure 5). For comparison, Pauly and Christensen [33] estimated that global fisheries on average consumed 8% of aNPP (range 2 to 35%), and that globally freshwaters average 24%. The majority of the scenarios for the Tonle Sap fall above these values.

There are multiple potential reasons why the Tonle Sap fish production is high relative to the amount of aNPP, including possible overfishing depleting the spawning stock. However, evidence for widespread overfishing is limited. A second alternative is that intensive and indiscriminate fishing has shifted the food web to low trophic level species with high growth rates, increasing the overall efficiency of transferring carbon through the food web. There is theoretical evidence for this type of community shift with indiscriminant fisheries like the Tonle Sap, and some empirical evidence as well [44]. Third, the aquatic food web of the Tonle Sap, including fish, likely is incorporating allochthonous resources derived from the surrounding floodplain or translocated within the basin [4], [45]. Strong net heterotrophy supports the idea that a large amount of terrestrial/emergent organic matter is entering the aquatic environment. It is generally recognized that aquatic food webs in flood-pulse ecosystems like the Tonle Sap are subsidized by the floodplain through both direct consumption and by microbial pathways [4], [46] although quantifying the magnitude of this subsidy has been challenging. It is unknown how much of the total fish or fisheries production in the Tonle Sap is supported by allochthonous materials, but it could be a substantial fraction. Finally, in complex, heterogeneous systems like the Tonle Sap there can be multiple pathways for this carbon to enter the aquatic food web and support fish production, including consumption of heterotrophic bacteria by invertebrates (zooplankton, benthic insects) and subsequently fish. Fishes can also directly graze on vegetation or bacteria. A less well studied option for organic carbon to move into food webs is through methane production and consumption by methanogenic and methanotrophic bacteria respectively [47]. This pathway may be particularly important in environments with variable dissolved oxygen and strong redox gradients such as the Tonle Sap. Significant carbon transfer through multiple pathways would enhance the overall productivity of the system as well as allow for a diversity of resource acquisition strategies within the community.

There is very limited information on food web dynamics for the Tonle Sap, particularly at the base of the food web. The role of microbial processing of organic matter contributing to secondary production is completely unexplored for this ecosystem, yet, based on our results, likely exceptionally important to maintenance of fisheries. A more robust understanding of potential fish production will require identifying and quantifying the full set of ecological resource flows (autochthonous vs. allochthonous, aerobic vs. anaerobic) to fish. The work presented here is an essential first step in elucidating these connections.

### Conclusions

The data presented here are the first published estimates of aquatic primary production and ecosystem respiration for the Tonle Sap and the Mekong River system. The majority of dissolved oxygen data from the basin consists of infrequent “spot samples” that provide little insight into biogeochemical dynamics and effects of environmental change [16]. Our results confirm the flood-pulse hydrology and interaction between the aquatic environment and the floodplain as a major driver of ecosystem function in the Tonle Sap, both with respect to primary production and heterotrophic respiration. The Tonle Sap ecosystem is in many ways defined by its flood-pulse hydrologic regime and some of the most critical ecosystem functions people depend upon are tied to this process. This includes the transfer of terrestrial material to the aquatic environment that supports fish production through direct consumption or the microbial loop; both pathways are likely to be severely impacted if future dams modulate the lake’s hydrologic regime [11], [48]. Mainstem and tributary dams could also alter the *in situ* primary productivity of the lake and floodplain, yet the likely net effect is currently unknown. It is possible that aNPP per unit area will increase with reduced sediment and higher light conditions for autotrophs, or decrease due to reduced nutrient inputs with flooding. Better predictions as to how proposed upstream dams will impact the Tonle Sap ecosystem and the fishery specifically will require a better understanding of how the flood-pulse drives recruitment of fishes to exploitable size classes, *in situ* NPP and loading of allochthonous organic matter, and the dominant pathways of energy flow within the aquatic food web. Ultimately this information is essential for the effective management of a resource that supports the livelihoods of millions of people.

## References

[1] Lindeman RL (1942) The trophic-dynamic aspect of ecology. Ecology 23: 399–417.

[2] Vannote RL, Minshall GW, Cummins KW, Sedell JR, Cushing CE (1980) River continuum concept. Can J Fish Aquat Sci 37: 130–137.

[3] Schindler DE, Scheuerell MD (2002) Habitat coupling in lake ecosystems. Oikos 98: 177–189.

[4] Junk JW, Bayley PB, Sparks RE (1989) The flood pulse concept in river flood plain systems. Can Spec Publ Fish Aquat Sci 106: 110–127.

[5] Odum HT (1956) Primary production in flowing waters. Limnol Oceanogr 1: 102–117.

[6] Staehr PA, Testa JM, Kemp WM, Cole JJ, Sand-Jensen K, et al (2012) The metabolism of aquatic ecosystems: history, applications, and future challenges. Aquat Sci 74: 15–29.

[7] Junk WJ (1997) The central Amazon floodplain: ecology of a pulsing system. Berlin; New York: Springer.

[8] Stone R (2011) Mayhem on the Mekong. Science 333: 814–818. 21835993 10.1126/science.333.6044.814

[9] Ziv G, Baran E, Rodríguez-Iturbe I, Levin SA (2012) Trading-off fish biodiversity, food security, and hydropower in the Mekong River Basin. Proc Natl Acad Sci U S A 109: 5609–5614. 22393001 10.1073/pnas.1201423109 PMC3326487

[10] Lauri H, de Moel H, Ward PJ, Räsänen TA, Keskinen M, et al (2012) Future changes in Mekong River hydrology: impact of climate change and reservoir operation on discharge. Hydrol Earth Syst Sci 16: 4603–46199.

[11] Kummu M, Sarkkula J (2008) Impact of the Mekong River flow alteration on the Tonle Sap flood pulse. Ambio 37: 185–192. 18595273 10.1579/0044-7447(2008)37[185:iotmrf]2.0.co;2

[12] Johnston R, Kummu M (2012) Water resource models in the Mekong basin: a review. Water Resour Manag 26: 429–455.

[13] Kummu M, Lu XX, Wang JJ, Varis O (2010) Basin-wide sediment trapping efficiency of emerging reservoirs along the Mekong. Geomorphology 119: 181–197.

[14] MRC (2008) Annual Report 2008. Vientiane, Lao PDR: Mekong River Commision.

[15] Hortle KG (2007) Consumption and the yield of fish and other aquatic animals from the Lower Mekong Basin. VientianeLao PDR: Mekong River Commission. 87 p.

[16] Irvine KN, Richey JE, Holtgrieve GW, Sarkkula J, Sampson M (2011) Spatial and temporal variability of turbidity, dissolved oxygen, conductivity, temperature, and fluorescence in the lower Mekong River-Tonle Sap system identified using continuous monitoring. Intl J River Basin Manag 9: 151–168.

[17] Sarkkula J, Koponen J, Hellesten S, Keskinen M, Kiirikki M, et al.. (2003) Modelling Tonle Sap for Environmental Impact Assessment and Management Support (draft final report).

[18] GW Holtgrieve, unpublished data.

[19] Lamberts D (2001) Tonle Sap fisheries: a case study on floodplain gillnet fisheries in Siem Reap, Cambodia. BangkokThailand: FAO Regional Office for Asia and the Pacific. 133 p.

[20] Holtgrieve GW, Schindler DE, Branch TA, A'Mar ZT (2010) Simultaneous quantification of aquatic ecosystem metabolism and reaeration using a Bayesian statistical model of oxygen dynamics. Limnol Oceanogr 55: 1047–1063.

[21] Wanninkhof R (1992) Relationship between wind-speed and gas exchange over the ocean. J Geophys Res-Oceans 97: 7373–7382.

[22] Francis R, Shotton R (1997) ''Risk'' in fisheries management: a review. Can J Fish Aquat Sci 54: 1699–1715.

[23] Hilborn R, Mangel M (1997) The Ecological Detective: Confronting models with data; Levin SA, Horn HS, editors. Princeton, New Jersey: Princeton University Press.

[24] Batt RD, Carpenter SR (2012) Free-water lake metabolism: addressing noisy time series with a Kalman filter. Limnol Oceanogr: Meth 10: 20–30.

[25] Ho DT, Wanninkhof R, Schlosser P, Ullman DS, Hebert D, et al.. (2011) Toward a universal relationship between wind speed and gas exchange: Gas transfer velocities measured with (3)He/SF(6) during the Southern Ocean Gas Exchange Experiment. J Geophys Res-Oceans 116, C00F04, doi:10.1029/2010JC006854.

[26] JAGS website. Available: http://mcmc-jags.sourceforge.net/. Accessed 2012 Feb 28.

[27] Su Y, Yajima M (2012) R2jags: A Package for Running jags from R. R package. R package version 0.03–06 ed.

[28] Denwood MJ (2011) runjags: Run Bayesian MCMC Models in the BUGS syntax from Within R. R package version 0.9.9–2 ed.

[29] Koponen J, Lamberts D, Sarkkula J, Inkala A, Junk JW, et al.. (2010) Detailed Modelling Support Project: Primary and Fish Production Report. HelsinkiFindland: Finnish Environment Institute. 104 p.

[30] Lamberts D, Koponen J (2008) Flood pulse alterations and productivity of the Tonle Sap ecosystem: A model for impact assessment. Ambio 37: 178–184. 18595272 10.1579/0044-7447(2008)37[178:fpaapo]2.0.co;2

[31] Likens GE (1975) Primary production of inland aquatic ecosystems. In: Lieth H, Whittaker RH, editors. Primary producitvity of the biosphere. New York: Springer-Verlab. 185–202.

[32] Melack JM (1976) Primary production and fish yields in tropical lakes. Trans Am Fish Soc 105: 575–580.

[33] Pauly D, Christensen V (1995) Primary production required to sustain global fisheries. Nature 374: 255–257.

[34] Deap L, Ly S, van Zalinge NP (1998) Catch statistics of the Cambodian freshwater fisheries. Phnom Penh: Mekong River Commission.

[35] Froese R, Pauly D (2003) FishBase. Available: http://www.fishbase.org/. Accessed 15 August 2012.

[36] Solomon CT, Bruesewitz DA, Richardson DC, Rose KC, Van de Bogert MC, et al (2013) Ecosystem respiration: drivers of daily variability and background respiration in lakes around the globe. Limnol Oceanogr 58(3): 849–866.

[37] van Zalinge NP (2002) Update on the status of the Cambodian inland capture fisheries sector with special reference to the Tonle Sap Great Lake. Catch and Culture. Phnom Penh: Mekong River Commission. 1–5.

[38] Lamberts D (2006) The Tonle Sap Lake as a productive ecosystem. Int J Water Resour D 22: 481–495.

[39] Rich PH, Wetzel RG (1978) Detritus in lake ecosystems. Am Nat 112: 57–71.

[40] Van de Bogert MC, Carpenter SR, Cole JJ, Pace ML (2007) Assessing pelagic and benthic metabolism using free water measurements. Limnol Oceanogr: Meth 5: 145–155.

[41] Alin SR, Johnson TC (2007) Carbon cycling in large lakes of the world: A synthesis of production, burial, and lake-atmosphere exchange estimates. Glob Biogeochem Cycle 21, GB3002, doi:10.1029/2006GB002881.

[42] Ohtaka A, Watanabe R, Im S, Chhay R, Tsukawaki S (2010) Spatial and seasonal changes of net plankton and zoobenthos in Lake Tonle Sap, Cambodia. Limnology 11: 85–94.

[43] Wetzel RG (2001) Limnology: lake and river ecosystems. San DiegoCaliforniaUSA: Academic Press. 1006 p.

[44] K. McCann, personal communication.

[45] Hecky RE, Fee EJ, Kling HJ, Rudd JWM (1981) Relationship between primary production and fish production in Lake Tanganyika. Trans Am Fish Soc 110: 336–345.

[46] Hand RT (2002) System Structure, Natural History, Dynamic Modeling and Adaptive Management of The Mekong Watershed’s Tonle Sap-Great Lake, Cambodia: Ph.D. thesis, University of Maryland.

[47] Sanseverino AM, Bastviken D, Sundh I, Pickova J, Enrich-Prast A (2012) Methane carbon supports aquatic wood webs to the fish level. PLoS ONE 7(8): e42723 doi:10.1371/journal.pone.0042723 22880091 10.1371/journal.pone.0042723 PMC3413669

[48] Arias ME, Cochrane TA, Piman T, Kummu M, Caruso BS, et al (2012) Quantifying changes in flooding and habitats in the Tonle Sap Lake (Cambodia) caused by water infrastructure development and climate change in the Mekong Basin. J Environ Manage 112: 53–66. 22877742 10.1016/j.jenvman.2012.07.003

[49] Kummu M, Penny D, Sarkkula J, Koponen J (2008) Sediment: Curse or blessing for Tonle Sap Lake? Ambio 37: 158–163. 18595269 10.1579/0044-7447(2008)37[158:scobft]2.0.co;2

